# An Update on the Therapeutic Role of Alkylglycerols

**DOI:** 10.3390/md8082267

**Published:** 2010-08-05

**Authors:** Tommaso Iannitti, Beniamino Palmieri

**Affiliations:** 1 Department of Biological and Biomedical Sciences, Glasgow Caledonian University, Glasgow, UK; 2 Department of General Surgery and Surgical Specialties, Medical School and Surgical Clinic, University of Modena and Reggio Emilia, Modena, Italy

**Keywords:** alkylglycerols, alkoxyglycerols, bathyl, selachyl, chimyl, ether lipids

## Abstract

Scandinavian folk medicine used shark liver oil for the treatment of cancers and other ailments based on the rarity of tumors in sharks and their ability to resist infections. Shark liver oil is a source of alkylglycerols which have been studied as anti-cancer agents in several clinical trials. Moreover, alkylglycerols have been investigated for the treatment of radiation induced side effects and for their ability to boost the immune system. Several experimental studies have shown the ability of alkylglycerols to open the blood brain barrier to facilitate the access of therapeutic drugs to the central nervous system. This review covers the most important studies of alkylglycerols in both animals and humans.

## 1. Introduction

**Figure 1:**
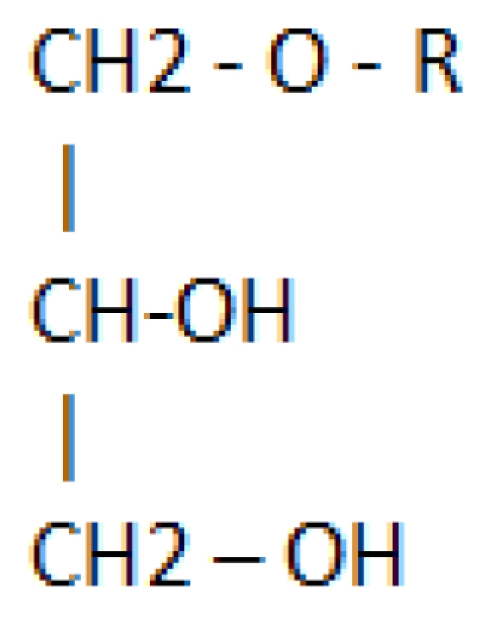
Chemical structure of alkylglycerols (AKG).

The shark is a member of the Elasmobranch subclass of fish that includes the ratfish (*Chimaera monstrosa*) and the dogfish (name used to designate a variety of shark species). Virtually all species of sharks are known to have an extraordinary resistance to the growth of tumors and infections. Several reports have indicated an extremely low incidence of cancer in sharks or even that no cases of cancer in sharks have been recorded [1,2] but a review performed by Ostrander and his colleagues identified a list of solid tumors found in sharks [3]. The emerging hypothesis is that *n*-3-polyunsaturated fatty acids (PUFA) and other shark liver oil (SLO) components may exert anti-carcinogenic effects [1]. SLO contains both alkylglycerols (AKG) and squalene and is an ancient remedy among the fishermen along the west coast of Norway and Sweden. It has been used for wound healing, the treatment of irritations of the respiratory and alimentary tracts and lymphadenopathy. In 1922 Tsujimoto and Toyama [4] found AKG in SLO and Sir Robert Robinson, a Nobel laureate, first synthesized them in 1930 [5]. In natural sources, they are always found esterified with fatty acids. Structurally they are alkyl ethers of glycerol (Figure 1).

Brohult and Holmberg [6], using the unsaponifiable portion of different bone marrow fats as well as preparations containing esters of AKG in child leukemia observed a maturing effect on the white blood cells leading to experiments employing AKG in irradiation leucopenia [7]. In the early 1950s, Brohult performed experiments on children with leukemia. She used extracts isolated from calf marrow and observed that they were able to stimulate the production of white blood cells. These finding led, in 1963, to the publication of a thesis on AKG and their use in radiation treatment [8]. This work showed that, in patients with uterine cancer, a decrease in white cells and thrombocytes, which usually occurs during radiation treatment, is less pronounced if AKG are administered during this treatment. After that it was observed that the incidence of injuries following radiation therapy for carcinoma of the uterine cervix was significantly decreased when the patients were treated with AKG [9] and that the frequency of fistulas was reduced by 47% when AKG were administered prior to radiation treatment [10].

The principal AKG include chimyl (hexadecyl), batyl (octadecyl) and selachyl (octadecyl) ethers. Hallgreen *et al*. [11] reported that glycerol ethers occur in the tissues in the form of diesters and alkyl acyl phosphatides. 1-*O-*Alkylglycerols and 1-*O-*(2-methoxyalkyl) glycerols were isolated from the neutral lipids and phospholipids of human colostrums, human milk, cow’s milk, sheep’s milk, human red bone marrow, red cells, blood plasma, and a uterine carcinoma (Table 1).

The authors found that: (1) human colostrum has a higher content of unsubstituted glycerol ethers in the neutral lipids than human milk; (2) human milk contains nearly 10 times more unsubstituted glycerol ethers than cow’s milk and twice as much as sheep’s milk; (3) the highest percentage of unsubstituted glycerol ethers in neutral lipids was found in the human red bone marrow and the uterine carcinoma; (4) the methoxy substituted glycerol ethers were both found in the neutral lipids and in the phospholipids of all the tissues studied but only in trace quantities; (5) glycerol ethers with 16 and 18 carbon atoms in the long hydrocarbon chains (16:0 chimyl, 18:0 batyl and 18:1 selachyl alcohol) are the principal components of both the unsubstituted and the 2-methoxy-substituted glycerol ethers; (6) a poly-unsaturated methoxy substituted glycerol ether, 1-*O-*(2-ethoxydocosahexaenyl-1) glycerol, was found in the neutral lipids and phospholipids of red blood cells (first found in Greenland SLO [14]). The authors have also reported several studies on the clinical effectiveness of glycerol ethers: (1) batyl alcohol raises the erythrocyte count of both normal rats and those poisoned with benzene; (2) optically active and racemic batyl alcohol stimulate erytrhopoesis, thrombopoesis and granulopoesis; (3) chymil alcohol stimulates haemopoesis; (4) selachyl alcohol has no haemopoetic activity; (5) a high level of glycerol ethers was found in a variety of transplantable tumors in animals and in human tumors; (6) 2-methoxy substituted glycerol ethers has antibiotic activity and inhibits the dissemination and growth of several experimental tumors in mice.

SLO is also rich in squalene, a triterpene that is an intermediate in cholesterol biosynthesis. We can also find it in olive oil, palm oil, wheat-germ oil, amaranth oil, and rice bran oil [15]. Squalene is the main component of skin surface polyunsaturated lipids as an emollient and antioxidant, and has hydration and antitumor activities; it also finds application in topically applied vehicles such as lipid emulsions and nanostructured lipid carriers [15]. 1-*O-*Alkylglycerols are naturally occurring ether-lipids, present in human or cow’s milk and in hematopoietic organs, such as bone marrow, spleen and liver [11,16]. SLO is rich in AKG and squalene, but contains relatively low amounts of *n*-3-PUFA. AKG may control immune response possibly through modification of platelet activating factor (PAF) and diacylglycerol (DAG) production. Squalene enhances antigen presentation and induction of the inflammatory response. Moreover, AKG and squalene have antitumor activity that may be based on different mechanisms, *i.e.*, induction of apoptosis of neoplastic cells, suppression of signal transduction, inhibition of angiogenesis and promotion of transmembrane transport of cytotoxic agents. SLO has been found to be useful in the treatment of conditions resulting from an inadequate immune response, and in adjunctive treatment of several types of cancer [17].

It has been shown that AKG can significantly reduce the injuries due to radiation toxicity, enhancing the overall survival rate and survival time in irradiated uterine cervical cancer patients [18]. Moreover, SLO enriched diets administered to rats with ischemic heart disease and hypertension, improve clinical symptoms, anthropometric levels, lipidemic profile and immunological status [19]. Nowicki *et al*. [20] emphasized the protective action of SLO from bacterial and fungal infections recommending it for patients suffering from atopic dermatitis.

Marigny *et al*. [21] cultured endothelial cells in the presence of 1-*O-*alkylglycerols resulting in inhibition of calcium ionophore and phorbol-12-myristate-13-acetate (PMA) increased endothelial permeability. This effect was associated with the production of an ether analogue of DAG described as an inhibitor of DAG-induced protein kinase C activation.

## 2. Review Criteria

We have searched Medline for studies involving the use of alkylglycerols with the keywords alkylglycerols, alkoxyglycerols, bathyl, selachyl, chymil and ether lipids. The results are categorized according to the way these compounds affect cancer, immunity, the blood brain barrier, bacteria and fungi, plasmalogens and radiation therapy.

## 3. Alkylglycerols Studies

### 3.1. Alkylglycerols and immunity

AKG and alkyl lysophospholipids significantly activate cytotoxic macrophages leading to enhanced Fc-receptor mediated phagocytosis and increase humoral immune response and delayed hypesensivity reaction [22]. AKG have been shown to stimulate hematopoiesis, erythropoiesis, thrombocytosis and granulocytosis in animals [23,24].

Sy *et al*. [25] designed a study to assess the effects of dietary supplementation with AKG in the range of 10, 50 and 250 ppm (chymil, batyl and selachyl glycerols solubilized in corn oil in the proportion of 30% chimyl, 28% batyl, and 42% selachyl glycerol to resemble the AKG composition in human milk) in lactating rats (Sprague-Dawley female rats, 8–10 weeks old) on AKG levels in milk and development of certain immune responses in the pups. Concentrations of AKG in milk from the dams fed AKG were significantly greater than those of the controls. Peripheral blood granulocytes were significantly elevated in pups from the dams fed AKG, but there were no differences in peripheral blood lymphocyte numbers. Plasma levels of immunoglobulins were significantly greater for IgG and IgM in pups from the dams fed AKG than in the control pups. This study shows that the murine milk contains glyceryl ether lipids in amounts comparable to those in human milk; increasing the levels of AKG in murine milk, the number of granulocytes in peripheral blood and plasma level of immunoglobulins, namely IgM was significantly increased in suckling pups. This study suggests that AKG in murine milk play a role in the development of immune response in newborn rat pups.

Homma *et al*. [26] treated a mixture of mouse (female BALB/c mice, aged 7–12 weeks) peritoneal non-adherent and adherent cells for 30 min with 50 ng dodecylglycerol (DDG)/mL in 10% foetal calf serum (FCS) supplemented RPMI-1640 medium. They observed a greatly enhanced ingestion of IgG-coated target cells but not IgM-coated target cells with complement. DDG treatment of adherent cells (macrophages) alone did not enhance ingestion activity of macrophages. This may be related to a signal factor(s) for macrophage activation which is transmitted from non-adherent cells to adherent cells during the brief DDG treatment period. Peritoneal cells were treated with 50 ng DDG/mL of a serum frec-01% egg albumin supplemented RPMI medium (EA) for 30 min and cultured in EA medium for 3 hours to try to identify the signal factor. A very small increase in ingestion activity of macrophages was observed. In contrast 30 min treatment of the peritoneal cells in FCS medium produced a greatly enhanced activation of macrophages. These observations suggested that a factor(s) contained in serum was required for activation of macrophages. Non-adherent cells were treated with 50 ng DDG in EA medium for 30 min., washed in PBS and cultured in FCS medium for 2 hours. The resultant conditioned medium of the DDG treated non-adherent cells was added to adherent cells and cultured for 3 hours. A greatly enhanced ingestion activity of macrophages was observed. However 3 hour cultivation of adherent cells with conditioned medium of the untreated non-adherent cells resulted in no enhanced ingestion activity of macrophages. These results indicate that the DDG treated non-adherent cells, if serum is present, generate a signal factor for macrophages to develop ingestion activity. The authors searched for the serum factor required for activation of macrophages through electrophoretic fractionation. FCS was electrophoresed in a starch block. Each fraction was added to DDG-treated splenic non-adherent cells in EA medium and cultured for 2 hours. The resultant conditioned medium was added to adherent cells for 3 hour cultivation prior to ingestion assay. Cultivation of adherent cells with the conditioned medium prepared with fraction no. 7 of electrophoresed serum produced a greatly enhanced ingestion activity of macrophages. With fraction no. 6, a lesser degree of macrophage activation was observed. No other fraction had significant macrophage activation activity. When analyzed by immunoelectrophoresis, fraction no. 7 (also no. 6 and 8) contained goat anti-bovine immunoreactable material, mainly in the α2-globulin region. This study showed that when peritoneal cells (mixture of non-adherent and adherent cells) are treated with 50 ng DDG/mL in FCS medium for 30 min and washed to remove residual DDG and nonadherent cells, the adherent macrophages develop a greatly enhanced ingestion activity after 3 hour cultivation. A rapid signal transfer from non-adherent cells to adherent cells must have occurred during a 30 min DDG treatment period. Enucleated cell ghosts of splenic non-adherent (B and T) cells by hypotonic shock in 1/10 PBS were prepared. The splenic non-adherent cell ghosts were treated with 50 ng DDG/mL in EA medium for 30 min, washed in PBS and incubated in α2-globulin (electrophoresed fraction no. 7) supplemented EA medium for 2 hours. After removal of the non-adherent cell ghosts, the medium was used for 3 hour cultivation of peritoneal adherent cells. A greatly enhanced Fc-receptor mediated ingestion activity of macrophages was observed. Therefore, they concluded that a serum component of α2-globulin fraction is rapidly modified by pre-existing membranous functions or enzymes of B and T cells to yield macrophage activating factor. Concluding, these results show that: (1) α2-globulin contains a factor required during cultivation of DDG-treated peritoneal cells for activation of macrophages; (2) one of the α2-globulin components is responsible for macrophage activation; (3) DBP is the vitamin D3 transport protein and its interaction with macrophages may be a prerequisite for macrophage differentiation and macrophage activation and serum DBP in α2-globulin serum fraction is a possible serum factor required for macrophage activation; (4) the conditioned media of individually DDG treated B and T cells are unable to activate macrophages after 3 hour cultivation of adherent cells suggesting that both cell types are involved in transferring activation signal to macrophages; (5) cultivation of adherent cells with a mixture of treated B cell conditioned medium and treated T cell conditioned medium produced no significant activation of macrophages emphasizing that signal transmission among non-adherent cells must have occurred.

Yamamoto *et al*. [27] observed that administration of small amounts (10–100 ng) of AKG to mice (female BALB/c mice, 7–12 weeks old) greatly enhanced macrophage activation for Fc-mediated ingestion activity at the 5th day post treatment. Moreover a low dose, 5 ng/kg DDG (100 ng/mouse) of animal body weight is the most effective dosage for macrophage activation. Administration of lower concentrations of a longer carbon chained AKG, *sn*-3-octadecylglycerol (batyl alcohol), to mice produced a similar activation of macrophages. *In vitro* treatment of cultured peritoneal cells, with a very low concentration (50 ng/mL) of DDG, activates macrophages in 2–3 hours; whena mixture of macrophages and nonadherent (B and T) cells was treated with DDG, a greatly enhanced Fc-mediated ingestion was observed at about 3 hour post treatment, suggesting that non-adherent cells contributed to the activation of macrophages. When a conditioned medium of DDG-treated B- or T-cells was admixed with macrophages and incubated for 3 hours, no significantly enhanced ingestion activity of macrophages was observed. Thus, exchange of signalling factor(s) among B- and T-cells was analyzed by transferring conditioned media of DDG-treated B- or T-cells to untreated T- or B-cells. When the resultant (treated B-cells–untreated T-cells) conditioned medium was admixed with untreated macrophages and incubated for 3 hours, a markedly enhanced Fc-mediated ingestion was observed (no significant increase in ingestion activity was found in macrophages incubated with the treated T-cell–untreated B-cell conditioned medium. The data reported in this study suggest that DDG treatment of B-cells triggers initiation of development of macrophage ingestion capacity. DDG-treated B-cells initiates macrophage activation processes by releasing and transmitting a signalling factor(s) to T-cells, and in turn the T-cells modify the factor or produce a new factor(s) capable of the ultimate stimulation of macrophages for ingestion capability. This study emphasizes that DDG application, as a chemotherapeutic agent, is due to potentiation of macrophage, *i.e.*, antigen-presenting cells. Treating the animals with these agents potentiates host immune systems against cancer activity and citotoxicity of malignant cells.

Yamamoto *et al*. [28] also observed that *in vitro* treatment of peritoneal cells (from female BALB/c mice, 7–12 weeks of age), with DDG (50 ng DDG/mL) in 10% FCS supplemented medium RPMI-1640 resulted in a greatly enhanced Fc receptor-mediated phagocytic activity of macrophages. This macrophage activation process requires a serum factor in the α2-globulin fraction. The interaction of a serum factor with non-adherent cells and modification of the serum factor by B and T cells are required for the *in vitro* activation of macrophages by DDG; purification of this serum factor by electrophoresis and by actin affinity chromatography shows to improve its precursor activity; a small amount of α-globulin fraction (0.05%, v/v) or purified human DBP (0.026 ng/mL) efficiently supports activation of macrophages; purified human DBP can be efficiently converted by DDG-treated nonadherent cells to a macrophage-activating factor. Conversion of DBP to the macrophage-activating factor in the absence of other serum proteins is very efficient; a minute amount as low as 26 pg/mL of purified human DBP can be converted to yield a sufficient amount of macrophage-activating factor to achieve activation of macrophages; higher doses of this serum factor yield their correspondingly large amounts of the macrophage activating factor, but an excess of the macrophage activating factor does not achieve a proportional enhancement of phagocytic activity but rather exhibits suppression of macrophage activation.

Mitre *et al*. [29] administered 32 g SLO/d to twelve pregnant and then lactating sows (from day 80 of pregnancy to weaning) to determine the effect on the growth and immune status of their offspring, compared with a control group. Sows were vaccinated against Aujeszky’s disease 21 days before term. Supplemented sows had higher levels of both erythrocytes and Hb in their blood, and higher concentrations of IgG, AKG and *n*-3 PUFA in their mammary secretions. In piglets from supplemented sows, leucocytes and IgG were higher. Supplementation with SLO resulted in an increase in Aujeszky antibodies in both blood and colostrum of sows after vaccination, together with an increase in Aujeszky antibodies in piglet blood. Dietary supplementation with SLO to gestating and lactating sows induced a positive effect on litter immune status associated with a modification of both lipid content and immune properties of the mother’s milk.

Pedrono *et al*. [30] studied the effect of natural AKG (AKG species varied according to the alkyl-chain length, with composition as follows: 14:0 = 0.7%, 16:0 = 9.1%, 16:1n-7 = 12.5%, 18:1n-9 = 68.1%, 18:1n-7 = 4.8% and other minor species (<0.1%) = 4.8%) purified from SLO on [^3^H]-serotonin release from rabbit platelets *in vitro*. [^3^H]-AKG (1 mM) were incorporated into platelet lipids and metabolised into phosphatidylcholine, phosphatidylethanolamine and phosphatidylinositol. AKG (10 mM) had no effect on spontaneous [^3^H]-serotonin release, partially inhibited platelet-activating factor and did not modify thrombin-induced release. This study shows that AKG inhibit partially and specifically PAF-induced platelet stimulation possibly interfering with PAF receptors.

Homma *et al*. [31] reported that a 30 min *in vitro* treatment of peritoneal cells (mixture of non-adherent and adherent cells from BALB/mice, 7–12 weeks of age) with 50 ng/mL of synthetic DDG resulted in greatly enhanced Fc-receptor-mediated ingestion activity of macrophages. In this study they observed that treatment of adherent cells (macrophages), alone with DDG, produced no significant enhancement of macrophage ingestion activity, implying that macrophage activation requires a contribution of non-adherent cells. This observation emphasized that a signal factor for macrophage activation has to be rapidly transmitted from non-adherent cells to adherent cells during the 30 min *in vitro* treatment of non-adherent and adherent cells. DDG treated non-adherent cells were found to generate a macrophage-activating signal factor. Studies with a serum free 0.1% egg albumin-supplemented RPMI 1640 medium revealed that a serum factor is essential for macrophage activation process. DDG-treated B cells rapidly transmit a factor to untreated T cells which yield the ultimate macrophage-activating factor. This signal transmission among these cells for the macrophage activation process is too rapid to allow time for synthesis of inducible gene products leading to the hypothesis that a serum factor is modified by the pre-existing function of DDG-treated B cells and further modified by the pre-existing function of untreated T cells to yield macrophage-activating factor. This hypothesis is confirmed by the demonstration that DDG-treated splenic non-adherent cell ghosts modify a serum factor to yield macrophage-activating factor.

Pedrono *et al*. [32] studied the role of AKG (prepared from *Centrophorus squamosus* SLO. AKG species varied according to the alkyl-chain length, whose composition was as follows: 14:0 = 0.7%, 16:0 = 9.1%, 16:1n-7 = 12.5%, 18:1n-9 = 68.1%, 18:1n-7 = 4.8% and other minor species = 4.8%) on the calcium signalling in Jurkat T-cells, an immortalized line of T lymphocyte cells with the ability to produce interleukin-2. AKG induced a dose-dependent increase of cytosolic calcium rate in Jurkat T-cells as a function of time (plateau reached after 20 min). When the experiments were performed in calcium-free buffer, the AKG response on the rise of intracellular calcium concentration [Ca^2+^]_i_ was wholly abolished compared with the one in calcium-containing buffer, suggesting that these ether lipids were able to induce a calcium influx by the opening of Ca^2+^ channels. This study showed that verapamil, an inhibitor of L-type VGCC, did not affect [Ca^2+^]_i_ rise stimulated by AKG. N-conotoxin, known to block N-type VGCC was shown to be able to abolish partially the AKG-activated entry of extracellular Ca^2+^ (up to 39% of the maximal calcium rise). AKG effect on the modifications in membrane potential (Vm) was assessed. Gramicidin, known to induce depolarisation, and AA, shown to provoke hyperpolarisation, were employed as controls. AKG evoked plasma membrane depolarisation three times higher than gramicidin. Addition of AA evoked hyperpolarisation in these cells. Addition of AA after AKG reversed the depolarisation induced by the latter. Moreover, successive addition of AKG induced additive increases in plasma membrane depolarisation in Jurkat T-cells. Concluding this study shows that AKG are able to increase [Ca^2+^]_i_ influx in human Jurkat T-cells possibly by modulating the permeability of calcium channels.

Tchorzewski *et al*. [33] proved that supportive treatment with SLO components normalizes complement level, natural killer (NK) cells activity and reactive oxygen intermediates production by peripheral blood leukocytes of people suffering from active form rheumatoid arthritis.

Acevedo *et al*. [34] evaluated the activation activity of AKG on the human histiocytic cell line U937 and the murine macrophagic cell line J774 to induce IL-12 and NO respectively, and the adjuvant activity of synthetic AKG to stimulate an antibody response (IgG2a-IgG1) against a soluble antigen (Ova). AKG stimulated the production of IL-12 and NO regardless of the carbon chain length; the levels of IL-12 induced by AKG appeared to be higher than LPS but lower than AFPs2 which is a powerful inducer of IL-12; AKG increased IgG levels compared with baseline levels induced by the administration of ova adsorbed to alum; the levels of anti-Ova IgG appeared to depend on the chain length of AKG; murine Th1 immune responses are associated with IgG2a production by B cells while Th2 responses are associated with IgG1 and IgE expression; high levels of IgG2a were also elicited when OVA were administered with AKG and AFPs2 as shown by the increase in the IgG2a/IgG1 ratio; all AKG elicited both Th1 and Th2 antibodies but AKG with longer chain length were associated with higher anti-Ova IgG2a production. This study shows that since AKG are able to induce a high level of IL-12, a key cytokine for the activation of Th1 responses, they could provide an environment for Th1 polarization. Synthetic AKG are effective adjuvants for the standardized antigen, Ova.

Pamblad *et al*. [35] designed a study to assess whether various AKG would initiate a functional response of human neutrophils or modify responses induced by a formyl peptide (fMLP) *in vitro*. The PAF was the most potent with regard to the ability to produce an oxidative response (assessed by cytochrome C reduction and/or chemiluminescence), followed by 1-*O-*hexadecyl-2-metoxy-glycero-3-phosphatidylcholine (ET-16-OCH_3_), a structurally modified ether lipid; Lyso-PAF, ET-18-OCH_3_, batyl- and chimyl alcohols exhibited only a weak activity; PAF was also the most efficient lipid conferring a rise of [Ca^2+^]_i_. ET-16-OCH_3_ and LysoPAF were less potent, although maximal [Ca^2+^]_i_ levels were similar to that of 0.1 pmoVl fMLP; the kinetics of the calcium responses were highly specific for each ether lipid; neutrophils treated with PAF or ET-18-OCH_3_ and subsequently stimulated by fMLP showed an enhancement of the oxidative response. The authors concluded that the previously described alkyl lipids are able to stimulate functional response in human neutrophils. PAF showed to be, on an equimolar basis, the most potent of tested ether lipids in conferring a secretory as well as a [Ca2+]i response. ET-16-OCH_3_ was also able to stimulate both functions. Some of the other phosphatidylcholine-containing ether lipids were associated with a rise of [Ca^2+^]_i_, but only with minute or no oxidative responses (LysoPAF > ET-18-OCH_3_ > M-lyso-PAF). CA, BA and M-BA (simple AKG) showed to be only weak stimulants for chemiluminescence. Apparently the phosphatidyl group in 3-position is essential for both calcium and oxidative responses (showed by LysoPAF and CA and BA). Moreover, the introduction of a methyl group in the 2-position is able to increase the tested biological activity (cf. ET-16-OCH3 and ET-18-OCH3 with LysoPAF), although the methyl group was less active than the acetyl group (cf. FT-16-OCH_3_ and ET-18-OCH3 with PAF). Insertion of a methoxy side-group on the fatty acid in the 1-position reduced the assessed biological activity (cf. LysoPAF with M-lysoPAF and BA with M-BA). An acetyl group in the 2-position was associated with greater efficacy, as demonstrated by comparisons of PAF with LysoPAF and OAG with MPG. Comparisons of PAF and IysoPAF with PPC and SPC, and of BA and CA with MPG, suggested that ether compounds were associated with enhanced biological activity. This study also showed that some ether lipids induced oxidative as well as [Ca^2+^]_i_ responses (PAF, ET-16-OCH_3_), whereas others mainly conferred a [Ca^2+^]_i_ rise (LysoPAF, ET-18-OCH_3_, M-lysoPAF). This suggested that a calcium rise is not sufficient by itself to activate the NADPH-oxidase, catalysing conversion of oxygen to superoxide ion. Since ether lipids have attracted interest as antitumour agents, ET-18-OCH_3_ has been advocated as a bone marrow purging substance, the intake of a mixture of CA, BA, M-BA and methoxy-substituted alkyl lipids, similar to M-lysoPAF has been shown to reduce the 5-year cervical cancer mortality and also the radiation-induced neutropenia in such patients. Ether lipids have also shown to increase blood concentrations of neutrophils and enhance antibody formation taken together.

Lewkowicz *et al*. [36] studied SLO supplementation with high doses (3.6 g) of squalene, 3.6 g of AKG and 750 mg of *n*-3-PUFA per day in 13 volunteers for 4 weeks. An increased response of neutrophils towards bacteria, an increased level of C4 component of complement in blood, the rise of total antioxidant status of serum, and the predominance of type 1 cytokine IFN-gamma, TNF-α and IL-2 production by peripheral blood mononuclear cells after SLO intake were observed. SLO supplementation markedly affected lipid metabolism and cholesterol balance too. The authors observed an increase of total cholesterol level from 182.92 ± 29.290 mg/dL before oil consumption to 224.46 ± 62.198 mg/dL after diet rich in oil and noted a decrease of HDL fraction. In all individuals metabolism of lipids normalized spontaneously after the end of the experiment. This study showed that the main effects of SLO are the result of the biological activity of squalene and 1-*O-*alkylglycerols. Moreover, they noted that the anti-inflammatory effects of *n*-3-PUFA did not manifest when taken together with high doses of squalene and AKG. This study shows that SLO supplementation in high doses is beneficial in bacterial, viral and fungal infections although patients with atherosclerosis or autoimmune diseases should avoid the consumption of high amounts of SLO.

Tchorzewski *et al*. [37] administered nine capsules of Biomarine 570 (an agent extracted from Greenland SLO) a day for 30 days to 10 healthy randomly chosen volunteers and observed that BioMarine intake increased the C1q level, the CD4/CD8 ratio from 1.3 to 1.8 and polarized Th1/Th2 lymphocyte cytokine secretion towards Th1; the modulation of reactive oxygen intermediates (ROI) production by neutrophils was also observed. BioMarine had no effect on CD4^+^CD25^+^ regulatory lymphocytes. This study showed no side effects proving that BioMarine is a safe, effective and an innate immunity supporting agent. This compound can be effectively used in people with disturbed immune system as harmless and effective agent normalizing the immune misbalance.

Le Blanc *et al*. [38] studied if Et-16-OCH_3_ and 1-0-hexadecyl-*sn*-glycerol (chimyl alcohol) possessed the ability to induce functional responses in platelets and chemiluminescence in neutrophils and whether they could modify the response induced by PAF. Et-16-OCH_3_ (1–100 μM) induced platelet aggregation but CA did not. Et-16-OCH_3_ induced platelet aggregation was abolished by pretreatment with the PAF receptor antagonist WEB 2086; CA had no effect on platelet aggregation induced by PAF, pretreatment with Et-16-OCH_3_ (0.1 PM or higher) significantly inhibited platelet aggregation induced by PAF, but it had no effect on aggregation caused by ADP, thrombin or PMA; a receptor binding study using radiolabelled [^3^H]WEB 2086 showed that Et-16-OCH_3_ exerts its actions through interaction with the PAF receptor; Et-16-OCH_3_ inhibited neutrophil chemiluminescence responses induced by PAF but not the reactions to PMA or a formyl peptide and 1 μM Et-16-OCH_3_ induced a rise in the [Ca^2+^]_i_ in platelets equal to that induced by PAF and it also had a calcium ionophore-like effect at 100 μM.

### 3.2. AKG and cancer

In an unpublished paper [39] Hajimoradi *et al*. investigated SLO activity as an immunomodulator. They used peritoneal macrophages from female inbred BALB/c mice, aged 8 to 10 weeks, to determine the *in vitro* effect of 100% pure natural arctic SLO, extracted from the Greenland Shark *Somniosus microcephalus* and observed that, with the increase of SLO concentration, nitric oxide (NO) production of macrophages increased with no multiplication of cells thus supporting the use of SLO as an adjunct therapy in the treatment of neoplastic disorders and as an immune booster in infectious diseases.

**Figure 3:**
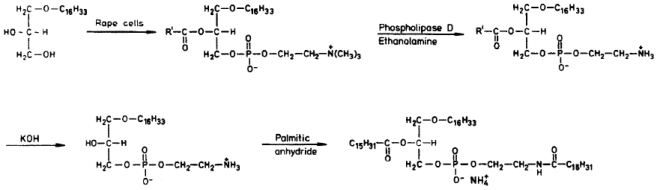
Preparation of l-hexadecyl-2-palmitoyl-*sn*-glycero-3-phospho-(*N*-palmitoyl)- ethanolamine by incubating photomixotrophic cell suspension cultures of rape (*B. napus*) with *rac*-1(3)-0-hexadecylglycerol followed by enzymatic and chemical reactions (C_16_H_33_ = hexadecyl; C_15_H_31_CO = palmitoyl; R’CO = acy1). Reprinted from “Biologically active ether lipids. Biotransformation of *rac*-1(3)-*O-*alkylglycerols in cell suspension cultures of rape and semisynthesis of 1-*O-*alkyl-2-palmitoyl-*sn*-glycero-3-phospho-(*N*-palmitoyl) ethanolamines, potent antitumor agents” [40], with permission from Elsevier.

Apte *et al*. [40] described the preparation of pure l-*O-*hexadecyl-2-palmitoyl-*sn*-glycero-3-phospho- (*N*-palmitoyl) ethanolamine (Figure 3) as well as of the corresponding 1-*O-*tetradecyl-, 1-*O-*octadecyl-, and 1-*O*[(*Z*)-9′-octadecenyl]-derivatives, each of which exhibits a strong antitumor effect in cultured neoplastic cells and in mice bearing MC 11 sarcomas.

Pedrono *et al*. [41] observed that orally AKG, administered to mice affected by Lewis lung carcinoma tumors, reduced metastasis dissemination by 64 ± 8%, whereas SLO effect was 30 ± 9% below control. Purified AKG also decreased significantly plasmalogen content in tumors, whereas SLO had no such effect. A 5-day treatment with AKG curtailed the presence in tumors of von Willebrand factor, a marker of endothelial cells suggesting an anti-angiogenic effect of AKG. This study shows that AKG decrease the growth, vascularization, and dissemination of carcinoma tumors in mice.

Skopinska-Rozewska *et al*. [42] tested SLO and fish liver oil together with arctic birch ashes in Balb/c mice after transplantation of syngeneic L-1 sarcoma observing that both the substances tested, alone or in combination, can significantly diminish cutaneous angiogenesis induced by tumor cells and tumor growth.

Hajimoradi *et al*. [43] assessed the anti-cancer effect of SLO [capsules contain 100% pure natural arctic SLO (350 mg/capsule), extracted from the liver of 101 Greenland sharks (*Somniosus microcephalus*). natural vitamin A, D and E (minor amount), AKG (35 mg/capsule) and ω-3 PUFA (42 mg/capsule)]. The injection (on 35 mice) of various concentrations of SLO (50, 10, 5, 2.5 and 0.1 mg/kg/day) indicated that SLO, at the doses of 50 and 10 mg/kg/day, had maximum enhancing effect on the delayed-type hypersensitivity (DTH) response after 48 hours; the percentage of CD8^+^ lymphocytes increased in the mice injected with SLO. SLO (10 mg/kg/day) injected intraperitoneally showed a decrease in the rate of tumor growth, but it was not statistically significant. After injecting 25 mice with SLO to evaluate the cytokine pattern in the tumor-bearing animals, a significant increase in IFN-gamma production was observed. These results underline that SLO may be administered for prophylaxis and treatment of disease in immunocompromised patients.

Wang *et al*. [44] investigated whether a methoxy-substituted alkylglycerol-1-*O*-(2-methoxy) hexadecyl glycerol (MHG) might promote a more benign or differentiated phenotype in three human colon cancer cell lines with different phenotypic properties (the moderately differentiated and growth factor responsive Moser, the growth factor unresponsive and malignant HT29, and the poorly differentiated and growth factor unresponsive HCT116). MHG inhibited the growth of all the three types of cells to a similar degree; 80% inhibition of cell growth was observed at 25 mM concentration; upon increasing the concentration of MHG no further increase in growth inhibition was observed; MHG upregulated the production of Carcino-Embryonales Antigen (CEA) in all the three cell lines; the Moser cells produced CEA in the amount of 5 ng/10^6^ cells. An increase in CEA production was observed when the cells were treated with either 10, 25, or 50 mM MHG. Treatment with 50 mM MHG upregulated CEA production to above 8 ng/10^6^ cells; 6) the HT29 cells produced slightly less CEA (4 ng/10^6^ cells) than the Moser cells and responded to all concentrations of MHG tested by increasing CEA production. Maximal CEA induction (8 ng/10^6^ cells) was observed when the cells were treated with 25 μM MHG; HCT116 cells produced the least amount of CEA (1–2 ng/10^6^ cells). Treatment of these cells with 25 μM MHG nevertheless upmodulated CEA production (3–4 ng/10^6^ cells); the treatment of the HT29 cells with MHG upregulated fibronectin production in a dose-dependent manner; the upregulation of fibronectin production was not observed in the Moser and HCT116 cells; the HCT116 cells were found to be the most aggressive, while the HT29 cells were intermediately aggressive and the Moser cells the least aggressive in terms of anchorage-independent growth and cellular invasion; MHG-treated cells showed a reduced propensity to grow in soft agarose in all three cell lines. A similar level of inhibition (36–37%) was observed for all cell lines when treated with 25 μM MHG, and a slightly higher level of inhibition (59%) was achieved for the HCT116 cells when treated with 50 μM MHG in comparison with the Moser (49%) and HT29 (44%) cells; MHG-treated cells showed a reduced ability to invade a matrigel matrix. The Moser cells did not respond to treatment with 25 μM MHG, while a slight reduction in cellular invasion was observed for the HT29 and HCT116 cells. Treatment of these cell lines with 50 μM MHG reduced the invasive capability of the HCT116, HT29 and Moser cells by 47, 35 and 19% respectively. This study shows that MHG is biologically active and is able to promote a more benign or differentiated phenotype in these colon cancer cells.

Krotkiewski *et al*. [45], using the plating efficiency method, assessed the effect of AKG on the plating efficiency of human ovarian carcinoma (OVP-10), mammary carcinoma (MCF-7), and prostate cancer (DU-145, PC-3 and PCa-2b) cell lines. The cells were exposed to Ecomer® SLO containing 20% AKG and 3% methoxyderivates in a dose of 0.1 mg/mL, up to a concentration corresponding to LD-50. The prostate cells from DU-145, PC-3 and PCa-2B showed a dramatic reduction in the colony number even after relatively small doses of 0.5 and 0.1 mg/mL medium; flow cytometry showed an increased percentage of apoptotic cells of ovarian and prostate carcinoma, while mammary carcinoma cells showed predominantly necrotic cells after exposure to Ecomer®.

This study shows a clear apoptosis/necrosis-inducing effect of Ecomer in three different cell lines of human prostate cancer, and in human mammary carcinoma cells line but no such effect has been observed in the human ovarian carcinoma cells.

Pedrono *et al*. [46] studied the effects of AKG (from *Centrophorus squamosus*; composition: 14:0 = 0.7%, 16:0 = 9.1%, 16:1n-7 = 12.5%, 18:1n-9 = 68.1%, 18:1n-7 = 4.8% and other minor species (<1%) = 4.8%) on the proliferation of basic Fibroblast Growth Factor (bFGF) stimulated endothelial cells. AKG were metabolized into alkyl-glycerophosphocholine and alkyl-glycerophosphoinositol, two ether analogues of phospholipids potentially involved in cell signalling; AKG were metabolized into several lipids that could influence signal transduction, namely alkyl-PA, alkyl-lysoPA and alkyl-acyl-Gro; AKG influenced endothelial cell growth, without any cytotoxic effects, and decreased the cell proliferation in a concentration and a time-dependent manner; they also reduced the stimulating effect of bFGF and the effects of 0.5, and 5 ng/mL bFGF on growth were completely suppressed after 72 h-treatment by 50 microM AKG. This study shows that bFGF greatly increases (+56% ± 15) the production of 1-*O-*alkyl-2-acyl-*sn*-glycerophosphate in AKG-treated endothelial cells, suggesting that the observed effects of AKG could be mediated through Phospholipase D activation. This may lead to new therapeutic perspectives.

Rait *et al*. [47] showed that anti-ras 3′-end conjugates of minimally phosphorothioate-protected oligonucleotides with 1-*O-*hexadecylglycerol (PPS-C16 ODN) retained the high sequence-specificity of PPS ODNs and provided maximal inhibition of Ras p21 synthesis with minimal toxicity even without the use of a cellular uptake enhancer. Moreover, treatment of T24, a radiation-resistant human tumor cell line that carries a mutant ras gene with anti-ras PPS-C16 ODN, results in a reduction in the radiation resistance of the cells *in vitro*. This study also demonstrated that the growth of RS504 (a human c-Ha-ras transformed NIH/3T3 cell line) mouse tumors is significantly inhibited by the combination of intratumoral injection of anti-ras PPS-C16 ODN and radiation treatment. These findings indicate the potential of the combination of antisense and conventional radiation therapy as a highly effective cancer treatment modality.

Reynolds *et al*. [48] observed that both the human prostate cancer LnCap and DU145 cell lines responded to the antiproliferative effect of MHG in a similar manner. In this study MHG inhibited cell growth with equal potency in these cell lines with an IC-50 value of 93 μM for LnCap, and 97 μM for DU145 while the IC-50 values for phenylbutyrate were 1.3 mM and 7.3 mM, respectively, for LnCap and DU145 cells. Compared to the DU145 cells the LnCap cells are at least five times more sensitive to the antiproliferative effect of PB, probably due to the differences in the biological phenotype of the two cell lines. LnCap cells showed to have a greater propensity to grow in soft agarose than the DU145 cells (2,500 *vs.* 1,500 colonies) and both MHG and PB (IC-50 concentrations) inhibited the growth of these cells in soft agarose. As much as over 50% inhibition was achieved for both LnCap and DU145 cells by PB and a lesser degree of inhibition was observed with MHG. MHG- and PB-treated cells showed a reduced ability to invade a matrigel matrix. Both the LnCap and DU145 cells invaded the matrigel to a similar extent. Invasion by PB-treated LnCap and DU145 cells was reduced, respectively, by approximating to 41 and 30% when compared to untreated control cells. Invasion of MHG-treated LnCap and DU145 cells was reduced, respectively, of about 25 and 9%. This study showed that MHG in μM concentrations also inhibits cellular proliferation, anchorage-independent growth and cellular invasion in the human prostate LnCap and DU145 cells. At equivalent IC-50 concentrations it was found that PB is more potent than MHG in inhibiting anchorage-independent growth and cellular invasion. However both MHG and PB inhibited the malignant properties of these prostate cancer cells but the concentrations required to achieve similar inhibitory effect were significantly different for these two agents.

According to previously described studies, the anticancer effect of AKG might be due to the property of activating macrophages, and increasing the cytokines such as IL-12 and IFN-gamma production. The recruitment and activation of macrophages, in fact, are acknowledged to be fundamental in the primary antitumor defence [49] while IFN-gamma, a T-cell derived lymphokine, inhibits a wide number of malignant cells, either directly counteracting cancer cells growth or through its immunomodulatory properties [50].

IL-12 induces the secretion of IFN-gamma from naive and activated T and NK cells, enhances the cytotoxic activity of NK cells, cytotoxic T lymphocytes, lymphokine activated killer cells and increases the proliferation of preactivated T cells and NK cells; the IL-12 ability to induce differentiation of Th1 cells, and to produce cell-mediated immunity enhancing cytokines, underlines how this cytokine plays a key role in promoting host defence and protection against cancer [51,52].

Another possible scenario, explaining AKG antitumoral activity, could be found in the accumulation of *O-*alkyl groups in tumoral cells which are not able to face the abnormal distribution of these glyceryl ether lipids [53] that is due to a low or absent expression of *O-*alkyl monooxygenase enzyme activity with a subsequent accumulation of lipid ethers in the tissue leading to cell death [54].

### 3.3. Alkylglycerols and blood brain barrier

The discovery of the blood brain barrier (BBB) can be attributed to the researcher Edwin Goldman who, in 1913, injected a dye directly into the brain, and realized that the dye failed to spread, suggesting that some sort of barrier was keeping it in. The BBB represents a major obstacle to the delivery of drugs to the central nervous system (CNS). The BBB consists of several barriers in parallel, with the two that are best described being the vascular BBB, consisting primarily of the capillary bed, and the blood-cerebrospinal fluid barrier, consisting primarily of the choroid plexus [55]. At both sites, the BBB is formed by a monolayer of cells that are cemented together by tight junctions and have other mechanisms that control or retard leakage of plasma into the CNS. The BBB accomplishes several roles: (1) blocking circulating substances from entering the CNS; (2) it facilitates and regulates the entry of many substances that are critical to the CNS function; (3) it secretes substances into the blood and the CNS. So it is able to influence the homeostatic, nutritive, and immune environments of the CNS and it regulates the exchange of informational molecules between the CNS and blood [56].

Erdlenbruch *et al*. [57] investigated the spatial distribution of intracarotid fluorescein sodium and intravenous lissamine-rhodamine B200 (RB 200) albumin in the brain of normal and C6 glioma-bearing rats (Male Wistar rats and male nude mice) after intracarotid co-administration of 1-*O-*pentylglycerol. The differential permeability of the BBB in the absence and presence of 200 mM 1-*O-*pentylglycerol was investigated in tumor-free rats (n = 19) and in rats bearing C6 gliomas (n = 12) using small and large fluorescence markers. The delivery of methotrexate (MTX) to the brain of tumor-free nude mice was evaluated in the absence and presence of 1-*O-*pentylglycerol (n = 12). In order to elucidate the mechanisms involved in the AKG-mediated BBB opening, intraluminal accumulation of fluorescein isothiocyanate (FITC) Dextran 40,000 was studied in freshly isolated rat brain capillaries using confocal microscopy during incubation with different AKG. Furthermore, 1-*O-*pentylglycerol-induced increase in delivery of MTX to the brain was evaluated in nude mice. Three microscopic evaluations showed a marked 1-*O-*pentylglycerol-induced extravasation of fluorescein and RB 200-albumin in the ipsilateral normal brain. In glioma-bearing rats, increased tissue fluorescence was found in both tumor tissue and brain surrounding tumor. Confocal microscopy revealed a time- and concentration-dependent accumulation of FITC–Dextran 40,000 within the lumina of isolated rat brain capillaries during incubation with 1-*O-*pentylglycerol and 2-*O-*hexyldiglycerol, indicating enhanced paracellular transfer via tight junctions. Intracarotid coadministration of MTX and 1-*O-*pentylglycerol (200 mM) in nude mice resulted in a significant increase in MTX concentrations in the ipsilateral brain as compared to controls without 1-*O*-pentylglycerol. In this study a strong increase in delivery of fluorescence markers of different molecular weight to both normal brain and brain tumors was demonstrated in rats by intracarotid coadministration of 1-*O-*pentylglycerol; an increased drug transfer across the BBB was also observed after intracarotid 1-*O*-pentylglycerol in nude mice. The permeabilizing effect of the AKG is mediated at least in part by enhanced permeability of the tight junctions and the ability of 1-*O-*pentylglycerol to increase the delivery of small and large compounds to normal brain and brain tumors is also mediated at least in part by enhanced permeability of tight junctions.

The same group [58] analyzed the transfer of MTX across the BBB in normal male Wistar rats to elucidate the effectiveness and structure activity relations of the most promising pentyl- and hexylglycerol derivatives. The effects were compared with BBB disruption using hypertonic mannitol or intracarotid infusion of bradykinin. Toxicity of the AKG has been studied in long-term experiments. The authors observed that: (1) apart from 1-*O-*pentyldiglycerol, all AKG induced a concentration-dependent increase in MTX delivery to the brain varying from 1.1 to more than 300-fold compared to intra-arterial MTX alone; (2) enhanced barrier permeability rapidly approached baseline values within 5 and 120 min at the latest; (3) mannitol 1.4 M resulted in very high MTX levels in the brain as observed using the highest concentrations of AKG; (4) intracarotid infusion of bradykinin had only a minor effect on the BBB; (5) using 1-*O-*pentylglycerol or 2-*O-*hexyldiglycerol, both cell culture experiments and long-term *in vivo* analyses including clinical, laboratory and histopathological evaluations revealed no signs of toxicity.

In summary, intracarotid short-chain AKG constitute a very effective and low toxic strategy for transient opening of the BBB to overcome the limited access of cytotoxic drugs to the brain. The Authors concluded that short-chain AKG show to increase the transfer of MTX to the brain and several instruments have been described to control the AKG mediated increase in barrier permeability. *In vitro* and *in vivo* assessment reveal that this new concept of BBB opening is associated with no toxic effects at therapeutic levels. Therefore intracarotid AKG represent a new therapeutic procedure to overcome the limited access of therapeutic agents to the CNS.

Erdlenbruch *et al*. [59] investigated a method to chemically open the BBB using intraarterial (i.a.) administration of AKG to increase the transfer of erucylphosphocholine (ErPC), a new derivative of the alkylphosphocholine (APC) family, into the brain. In contrast to hexadecylphosphocholine, the prototypical APC, ErPC can be administered intravenously without causing hemolysis. ErPC possesses a cell membrane-mediated mechanisms of action which is thought to be responsible for its ability to overcome the chemoresistance against standard anticancer agents frequently observed in high-grade gliomas. ErPC was administered to C6 glioma-bearing rats (Wistar rats weighing between 230 and 305 g which had had free access to a standard diet (Altromin) and tap-water during the whole experimental period) either as a single intracarotid bolus injection in the presence or absence of 1-*O-*pentylglycerol (300 mM) or as an intracarotid infusion in conjunction with bradykinin. Brain tissue concentrations were analyzed and compared to values obtained after intravenous ErPC treatment over 14 and 30 days (cumulative ErPC doses of 210 and 350 mg/kg, respectively. Increasing doses from 10 to 40 mg/kg, both single intravenous injection (i.v.) and single intracarotid bolus injection of ErPC, resulted in brain concentrations under the detection limit of 12 nmol/mg. In the first group receiving repeated i.v. ErPC injections (constant dose regimen; 25 mg/kg every 48 hours over 30 days; cumulative dose 350 mg/kg), an ErPC accumulation was found in the brain with tissue concentrations averaging 152 ± 53 pmol/mg, which exceeded serum levels by about 1.7-fold. Tissue levels of ErPC in liver and kidney were higher and those of fat tissue lower than the CNS levels. This study showed that ErPC treatment was well tolerated by the animals, the clinical and histological examinations showed no signs of ErPC-induced toxicity and the assessment of laboratory parameters revealed no changes during ErPC treatment. In the second group receiving i.v. ErPC treatment (saturation regimen), a rapid and more pronounced drug accumulation was achieved in both tumor tissue and surrounding tumor-free brain. ErPC concentrations averaged 264 pmol/mg in the tumor and 244 pmol/mg in the remaining tumor-free brain. Thus, even though the cumulative ErPC dose was significantly lower using the saturation regimen (210 mg/kg compared to 350 mg/kg given in the constant i.v. ErPC group), a significantly higher ErPC transfer into the CNS was achieved (P < 0.05). The higher initial ErPC doses were associated with attenuated weight gain or stagnant body weight, a clinical sign of ErPC-induced toxicity. However, laboratory and morphological examinations revealed no other signs of toxicity. The single i.a. ErPC injection in the presence of 1-*O*-pentylglycerol resulted in very high ErPC concentrations in brain tumor tissue. Compared to control animals receiving intra arterial ErPC without pentylglycerol (aqueous and ethanolic ErPC groups) and with those receiving intracarotid bradykinin, a marked increase in drug delivery to the brain tumor and to the ipsilateral and contralateral tumor-free brain was achieved. The increased access of ErPC to the brain, after coinjection with pentylglycerol, was also associated with significantly lower plasma levels of ErPC. The pentylglycerol-mediated BBB opening appeared to be more pronounced in the tumor tissue, as evidenced by the concentration difference between tumor (289 pmol/mg) and surrounding brain tissue (ipsilateral, 163 pmol/mg). Moreover this study reports that intra arterial bradykinin was not followed by an increase in ErPC concentrations in either tumor tissue or surrounding tumor-free brain.

Using ethanolic ErPC, there were higher ErPC concentrations in all brain regions than after the use of aqueous ErPC). Thus, ethanol itself induced an increase in BBB permeability and the ethanolic ErPC solution was therefore not suitable as a control to assess the pentylglycerol-associated effects at the BBB.

In summary, pentylglycerol-induced BBB opening dramatically increase the tissue concentrations of ErPC in the tumor and surrounding brain after i.a. injection; intracarotid bradykinin is ineffective; the ErPC levels measured in the CNS also exceed those obtained after long-term i.v. treatment; in the future AKG may represent a novel instrument to increase delivery of chemotherapeutic agents to brain tumors but also to more distant regions of the brain containing infiltrating and migrating cells that might be responsible for tumor recurrence; the selectiveness of the effect in favour of the tumor tissue reduces the potential for toxicity in the normal brain; intra arterial administration of ErPC in the presence of AKG is a promising new concept for brain tumor chemotherapy.

Erdlenbruch *et al*. [60] investigated the enhancement of the BBB permeability by i.a. administration of AKG in tumor-free and C6 astroglioma bearing rats. The antineoplastic agents cisplatin and MTX and the antibiotics vancomycin and gentamicin were selectively injected into the right internal carotid artery in the absence and presence of various alkylmono-, alkyldi-, and alkyltriglycerols. I.a. injection of CDDP, MTX, gentamicin, or vancomycin into the right internal carotid artery of rats in the absence of AKG resulted in low tissue concentrations of each drug with no regional differences between the right hemisphere, the left hemisphere and the cerebellum. Simultaneous administration with various AKG analogues showed an accumulation of the drugs in the brain, predominantly in the ipsilateral right hemisphere; using 1-*O-*pentylglycerol (0.3 M) the tissue concentrations in the ipsilateral hemisphere were increased 230-fold (MTX), 125-fold (CDDP), 15-fold (vancomycin) and 12-fold (gentamicin) compared to the injection in the absence of AKG (1E-H, n = 6 in each group); AKG effect on the blood-brain barrier was concentration dependent and increased with the chain length of the alkyl group; Heptyl- and octyl derivatives were highly potent even at low doses, resulting in high concentrations of the coinjected drugs in the ipsilateral hemisphere and also, to a lesser extent, in the contralateral hemisphere and cerebellum; enhancement of drug delivery to the brain was weakened by the high polarity of the diglycerol and, in particular, the triglycerol analogues; the administration of CDDP together with 1-*O-*butyl- or 1-*O-*pentylglycerol was responsible for a significant accumulation of CDDP in the right hemisphere, while the shorter 1-Opropyl-and the spheroid 1-*O-*tert-butylglycerol were ineffective; the 2-*O-*alkyl-isomers were less effective than the respective 1-*O-*alkyl-isomers probably due to a decrease in lipophilia of the molecules; separating the injection of AKG and MTX or CDDP by a time interval of 3 or 15 min led to an extensive attenuation or a complete loss (CDDP) of the enhanced barrier permeability but the effect of the AKG at the blood-brain barrier was reversible within a few minutes; hematological and serum analyses (sodium, potassium, calcium, glucose, total protein, aminotransferases, lactate dehydrogenase, bilirubin, and creatinine) revealed no acute toxic side effects of the monoglycerol analogues up to 0.3 M (hexylmonoglycerol was only injected up to 0.1 M); the diglycerol derivatives caused hemolysis at concentrations exceeding 0.1 M. 2-*O-*Heptyl-triglycerol and 2-*O-*octyltriglycerol were hemolytic exceeding concentrations of 0.075 and 0.05 M, respectively; coadministration of 1-*O-*pentylglycerol with MTX at the blood-tumor barrier to C6 rat astroglioma bearing rats showed that in the presence of 1-*O-*pentylglycerol (0.3 M) the concentrations of MTX were increased 18-fold in the tumor, 28-fold in the surrounding brain, 18-fold in the contralateral brain, and 19-fold in the cerebellum compared with MTX controls in the absence of pentylglycerol.

### 3.4. Alkylglycerols and plasmalogens

**Figure 4:**
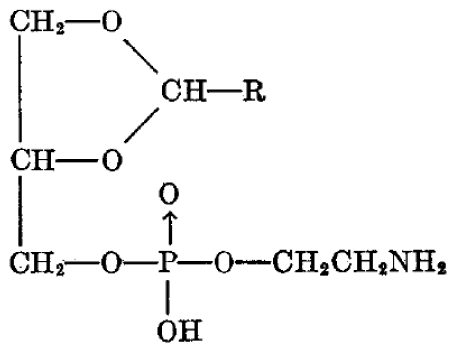
Plasmalogen acetal structure proposed by Feulgen in 1939 [61].

1-(1′-Alkenyl)-2-acyl glycerophospholipids and plasmalogens are the two types of phospholipids that may occur in biological tissue. Plasmalogens are characterized by a *Z*-double bond in the 1-alkenyl moiety next to the ether linkage. The exact percentage of plasmalogens varies from organ to organ as well as from species to species. In the heart over 25% of the phospholipids can be plasmalogens but they are present also in the liver, retina, brain and kidneys [61]. Plasmalogens were discovered in 1924 by Feulgen, whose studies leaded to the isolation in 1939 of a crystalline phospholipid-containing higher fatty aldehyde, glycerol phosphorus, and ethanolamine. Their proposed acetal structure has since served as a prototype of this class of lipids (Figure 4).

Myocardial sarcolemmal phospholipids are comprised predominantly of plasmalogen molecular species, and a growing body of evidence indicates that plasmalogen phospholipids may play a role in the pathophysiology of some heart diseases. Thus plasmalogens are believed to be involved in the sarcolemmal dysfunction associated with myocardial ischemia and reperfusion. It has been speculated that the distribution of plasmalogens in the heart might affect the bilayer stability, which may be instrumental in sarcolemmal destruction during ischemia and reperfusion. Membrane phospholipids are known to play a key role in myocardial ischemia reperfusion injury. Reperfusion of ischemic myocardium is associated with the loss of membrane phospholipids with the accumulation of lysophosphoglycerides and free fatty acids, especially arachidonic acid (AA). Mammalian hearts contain two types of phospholipids, diacyl and ether glycerolipids, also known as plasmalogens. A loss of plasmalogens occur in the pathogenesis of ischemic heart disease. Alkyl glycerols such as chimyl alcohol, can serve as a precursor for the formation of plasmalogens in the microsomes.

Maulik *et al*. [62] designed a study to examine whether supplementation of the heart with chimyl alcohol prior to ischemia could reduce the ischemia reperfusion injury. The study was performed in Sprague-Dawley rats weighing approximately 300 g. Their hearts were quickly removed and perfused by the Langendorff technique. The animals were divided into two groups: (1) the experimental group which received 50 pM chimyl alcohol prior to ischemia; (2) the control group which received 50 pM of saline. After 15 min of perfusion with or without the chirnyl alcohol, the heart was arrested at normothermia terminating the coronary flow. Coronary flow was significantly higher in the postischemic chimyl alcohol-treated heart as compared to the matched control; left ventricular developed pressure, during the reperfusion phase was also significantly better in the experimental group (9 ± 1 *versus* 13 ± 2 mm Hg); creatine kinase (CK) release from the postischemic heart was increased significantly from both the control and chimyl alcohol groups, but the amount of CK release was much lower in the treated group as compared to the control group (60 ± 10 *versus* 106 ± 9 IUII) after 30 min of reperfusion; the amount of malondialdehyde (MDA) in the postischemic heart was increased steadily in both groups, but the increase was significantly lower in the chimyl alcohol group (0.166 ± 0.008 *versus* 0.08 ± 0.002 nmol/mL); the peroxisomal catalase was significantly lowered in the postischemic heart (11.26 ± 0.8 *versus* 15.6 ± 0.35 nmol/min/mg protein); treatment with chimyl alcohol restored the catalase activity to the pre-ischemic baseline level (16.24 ± 0.67 nmol/min/mg protein). The authors concluded that reperfusion of ischemic myocardium may lead to the peroxisomal disorder. Chimyl alcohol can protect the ischemic heart from reperfusion injury probably by enhancing the plasmalogen synthesis. Moreover the MDA significantly lower increase in the postischemic heart in the chimyl alcohol treated group suggests that this AKG is able to reduce oxidative stress.

Schrakamp *et al*. [63] studied *de novo* plasmalogen biosynthesis measuring the incorporation of [1-^14^C]hexadecanol into the alkenyl side chain of ethanolamine and choline plasmalogens of cultured skin fibroblasts from patients with different peroxisomal or related diseases, from heterozygotes for Zellweger syndrome and infantile Refsum disease, and from controls. After [1-^14^C]hexadecanol incorporation plasmalogen biosynthesis was normal in fibroblasts from patients with X-linked drenoleukodystrophy, adrenomyeloneuropathy, Refsum disease, and X-linked chondrodysplasia punctata. About 45% and about 4.5% of total phospholipid radioactivity is present in these cases in the alkenyl chains of ethanolamine and choline plasmalogens, respectively; plasmalogen biosynthesis was found to be strongly impaired in fibroblasts of patients with neonatal adrenoleukodystrophy, infantile Refsum disease, Zellweger syndrome, and rhizomelic chondrodysplasia punctata; rhizomelic chondrodysplasia punctata is most severely deficient in plasmalogen biosynthesis (2.4 and 0.5% of radioactivity in alkenyl chains of ethanolamine and choline plasmalogens, respectively), followed by Zellweger syndrome, neonatal adrenoleukodystrophy, and infantile Refsum disease, in this order. Heterozygotes for Zellweger syndrome and infantile Refsum disease behaved like controls with respect to their plasmalogen biosynthetic capacity; no accumulation of alkylphospholipids in fibroblasts was found for any disease suggesting that the plasmalogen deficiency, reported before, is caused by impaired glycerol-ether bond formation in all these disorders; incorporation of l-*O-*[9,10-^3^H2]octadecylglycerol into ethanolamine and choline plasmalogens of fibroblasts of different peroxisomal diseases substantiated that the deficiency of plasmalogen biosynthesis in rhizomelic chondrodysplasia punctata, neonatal adrenoleukodystrophy, and infantile Refsum disease is at or before the introduction of the ether bond by peroxisomal enzymes.

Zoeller *et al*. [64] showed that supplementation of cultured human pulmonary arterial endothelial cells (PAEC) with *sn*-1-*O-*hexadecylglycerol (HG) resulted in an approximately twofold increase in cellular levels of plasmalogens. Exposure of unsupplemented human PAEC to hypoxia (pO2 ~ 20–25 mmHg) caused an increase in cellular reactive oxygen species (ROS) over a period of five days with a coincident decrease in viability. In contrast, HG supplemented cells survived for at least two weeks under these conditions with no evidence of increased ROS. Hypoxia resulted in a selective increase in the turnover of the plasmalogen plasmenylethanolamine. Human PAEC, with elevated plasmalogen levels, were also more resistant to H_2_O_2_, hyperoxia, and the superoxide generator plumbagin. This protection was seemingly specific to cellular stresses in which significant ROS were generated because the sensitivity to lethal heat shock or glucose deprivation was not altered in HG-treated human PAEC. HG, by itself, was not sufficient for protection; HG supplementation of bovine PAEC had no effect upon plasmalogen levels and did not rescue these cells from the cytotoxic effects of hypoxia. This study shows that the plasmalogen content of human PAEC can be enhanced. This is associated with an increased resistance to hypoxia as well as to the specific ROS or ROS generators. The use of HG to increase plasmalogen can have important implications for therapeutic use against hypoxia.

### 3.5. Alkylglycerols and radiation therapy

Hichami *et al*. [65] designed a study using a ^3^H-labelled or unlabelled natural AKG mixture [prominent alkyl chains were C18:1(9) (54–65%), C16:1(7) (5–15.5%), and C16:0 (5–10%)] to better understand the protection mechanism which is hypnotized to be mediated by AKG incorporation into pools of PAF precursor and subsequent modification of PAF biosynthesis. The authors investigated the incorporation of AKG into phospholipids of human leukemic monocyte-like THP-1 cells. Incubation of cells for 24 hours with [^3^H]AKG (10 μM) resulted in their incorporation into 1-*O-*alkyl-2-acyl-*sn*-glycero-3-phosphocholine and into 1-alkyl-2-acyl-*sn*-glycero-3-phosphoethanolamine with a total yield of 6.5%. The incorporation induced production of 1-*O-*[^3^H]alkyl-2-acetyl-*sn*-glycero-3-phosphocholine ([^3^H]PAF), which was increased after stimulation by the calcium ionophore A23187. HPLC analysis of the [^3^H]PAF molecular species indicated that the three major [^3^H]AKG were used for [^3^H]PAF synthesis in ratios similar to that of the mixture. Total production of biologically active PAF, as measured by the platelet-aggregation bioassay, was also increased by AKG incorporation in resting (120%) and in A23187-stimulated (159%) THP-1 cells. HPLC analysis of the [^3^H]PAF produced in the presence of [^3^H]acetate, confirmed that levels of PAF, but not of its 1-acyl analog, were increased by AKG incorporation in resting and stimulated cells. However, the rise in [^3^H]acetyl-PAF, which resulted mainly from C16:0 PAF, was reduced by about 50% in the presence of the PAF-receptor antagonist SR 27417, providing evidence that stimulation of total PAF synthesis was caused by the increase in the precursor pool and autocrine amplification of PAF-induced PAF production. Thus, the supplementation of THP-1 cells in culture with naturally occurring AKG led to the incorporation of AKG into ether-containing phospholipids which were subsequently used for PAF synthesis. Furthermore, AKG incorporation resulted in a significant rise in PAF production by THP-1 cells under resting and stimulated conditions. This study highlights that: (1) PAF species profile could be altered by the addition of, for example, C18:1 AKG; PAF exerts an important role in several pathophysiological situations and the possibility to modulate its synthesis could be an important tool to fight these pathologies; (2) Zellweger syndrome, a pathological condition, characterized by the absence of ultrastructurally detectable peroxisomes in patients’ tissues associated with the absence of ether-lipid synthesis, could be eased by a dietary supplementation with AKG.

Joelsson [66] designed a study to assess whether or not AKG, administered before and during in a prophylactic approach or only during in a therapeutic perspective, influence the incidence of injuries following radiotherapy for carcinoma of the uterine cervix. The Author used AKG preparation from the liver oil of the Greenland shark (concentrate containing 85% free AKG) administered orally in capsules containing 0.1 g. The total daily dosage amounted to 0.6 g. The patients with invasive carcinoma of the uterine cervix were assigned to one of the following groups: (1) patient receiving AKG prophylactically (during 7 days before and during the treatment period as well as for 1–3 months after the completion of radiotherapy); (2) patients receiving AKG only during the period of radiotherapy plus non-prophylactic administration for 1–3 months thereafter; (3) patients receiving radiotherapy solely. In this study the injuries, due to the combination of radiation tissue damage and residual and or recurrent tumor growth, were classified as complex injuries (C-injuries) and have been considered in addition. The sum of the injuries (R-injuries + C-injuries) was defined as the total number of injuries (I-injuries). The injuries were classified as: (1) grade I: injuries producing mild subjective symptoms accompanied by minimal objective changes in the mucosa of the organ. These injuries were considered as radiation reactions rather than real injuries and had consequently been omitted; (2) grade II: injuries which are composed of moderately to severe objective changes, such as areas of necrosis, ulcers or moderate stenosis; (3) grade III: bladder and ureter injuries comprising fistulas, and rectal and intestinal injuries comprising stenoses to such an extent that colostomy has been required; (4) grade IV: rectal and intestinal fistulas. Injuries which appeared within three months of treatment had been excluded and those injuries which were not clearly related to the radiation treatment or to tumor growth were also omitted. The authors, after the supplemention with AKG, observed that: (1) the incidence of total grade II injuries (I-injuries, grade II) was 9% in the prophylactic group and 24% in the control group (*i.e.*, a reduction of 60% was observed); (2) the incidence of grade II radiation injuries (R-injuries, grade II) was considerably reduced while no effect was observed on radiation injuries of grade III and IV; (3) the complex injuries of all grades were markedly reduced; (4) in the non-prophylactic group only the radiation injuries were reduced while the incidence of the complex injuries remained the same. The Author paid particular attention to the effect of AKG on the incidence of fistulas following radiation therapy (bladder injuries of grade III and rectal injuries of grade IV together constituted the fistulas). The author reported that: (1) the total number of fistulas (I-injuries grade III and IV, bladder and rectum) was considerably lower in the prophylactic group than in the control group (6.2% compared with 11.6%); (2) the fistulas, belonging to the complex injury group (C-injuries grade III and IV, bladder and rectum), had a low figure of to 2.9% compared with 7.3%; (3) the pure radiation fistulas (R-injuries grade III and IV, bladder and rectum) but not the fistulas of complex origin were decreased in number in the non-prophylactic group.

In summary an inhibition of tumor growth and a decrease of the number of both radiation and complex injuries was observed when AKG had been administered prior to radiation treatment of patients affected by cancer of the uterine cervix. In particular this study highlights that prophylactic administration of AKG markedly reduced fistulas proving the potential clinical importance of AKG as a complement to conventional radiation therapy in cancer treatment.

### 3.6. Antibacterial and antifungal activity of dodecylglycerols

Lipids, particularly fatty acid esters of polyhydric alcohols, have been widely reported to be antimicrobial agents. For a long period the monoglyceride dodecanoylglycerol (monolaurin) was known to be the most potent [67–68], but more recently DDG, the corresponsing AKG ether, has shown a considerably higher efficacy, probably due to a greater metabolic and chemical stability of ether bonds compared to esters.

DDG has been reported to exert its antibacterial activity by the activation or release of proteases which convert the autolytic enzyme autolysin from a latent inactive form to an active state. DDG was also found to be able to act by inhibiting the synthesis of the peptidoglycan in bacterial cell walls. Moreover its antifungal activity has been proved against several species of Candida and Cryptococcus [69]. Here we are going to summarize several clinical trials involving the use of DDG to test its antibacterial and antifungal properties.

Brisette *et al*. [70] used *Streptococcus mutans* BHT to study the response to DDG since the ether has a profound growth-inhibitory effect without inducing cellular lysis. *S. mutans* BHT is tolerant to penicillin and several other cell wall inhibiting antibiotics and responds to penicillin stimulating lipid and lipoteichoic acid synthesis and excretion. Growth-inhibitory concentrations of racemic *sn*-l(3)-dodecylglycerol inhibited the incorporation of [^14^C] glycerol into lipids and lipoteichoic acid of *Streptococcus mutans* BHT and altered the per cent composition of the glycerolipids; increases in phosphatidic acid and diphosphatidylglycerol (at the expense of phosphatidylglycerol) contributed the most to the change in lipid composition. No cellular lysis occured under these conditions; radioactive racemic *sn*-1(3)-dodecylglycerol is readily taken up by the cell and is metabolized primarily to lysophosphatidic acid and phosphatidic acid with smaller amounts converted to phosphatidylglycerol and diacylglycerol; the accumulation of phosphatidic acid and the loss of viability responded in parallel to different concentration of DDG; an increase in phosphorylcholine cytidylyl-transferase was also observed; this increase united to the the increase in phosphatidic acid suggested a possible impairment in the synthesis of CDP-diacylglycerol.

In summary, DDG (10 and 20 μg/mL) is able to inhibit glycerolipid and lipoteichoic acid synthesis from glycerol in *S. mutans* BHT basing on the observation that in cultures pre-equilibrated with [^3^H]glycerol or [^14^C]glycerol, DDG inhibited the incorporation of radioactive label into the glycerolipids. In the presence of 217 μM glycerol, DDG in a concentration of 38.4 μM (10 μg/mL) is readily taken up, metabolized, and inhibits lipid synthesis at the CDP-diacylglycerol synthetic step. It, in fact, inhibits the uptake and incorporation of [^14^C]glycerol into lipid. DDG functions as a metabolic effector, in fact DDG is an effective antibacterial agent at low concentrations (only a few μg/mL) and is strengthened by the present finding that DDG is metabolized, to lyso- and phosphatidic acid compounds. Furthermore, the accumulation of phosphatidic acid, both ether-containing and the natural difatty ester, suggests that there is a metabolic block at the CDP-diacylglycerol synthetase step. This metabolic effect is observed in the absence of any cellular lysis in *S. mutans* BHT. This, then, is the third known mechanism (of possibly many) by which DDG can interfere with normal bacterial metabolism. The other two are that it stimulates a proteinase (in *S. faecium*) which activates an autolysin, and it inhibits peptidoglycan synthesis. One or both of these metabolic changes are probably triggered by a disturbance induced by DDG to the hydrophobic environment of cellular membrane. A final conclusion is that DDG is bactericidal in *S. mutans* BHT. Substantial decreases of viability correlate closely with the accumulation of phosphatidic acid and a decreased energy profile. Moreover DDG shows to be able to kill the penicillin-tolerant bacterium, *S. mutans* BHT, proving to be valuable in combating tolerant bacteria where antibiotic tolerance has been correlated with an increased lipid content.

Weber *et al*. [71] investigated the use of orally administered rac-1-*O-*[l′-14C]dodecylglycerol in mice (female NMRI mice weighing 20–25 g). The substrate was rapidly absorbed in the intestine and then incorporated into ether glycerolipids of various organs, and tissues in high proportions; in intestine and liver large amounts of rac-l-*O-*[l′-14C]dodecylglycerol were catabolized by oxidative cleavage of the ether bond followed by degradation of the radioactive cleavage product, *i.e.*, lauric acid, to water-soluble metabolites that were excreted in the urine at a fast rate; after feeding of a rac-1–0-dodecylglycerol-containing diet (1 g rac-1-*O-*dodecylglycerol/kg body weight × day) given over a period of 4 weeks the author did not observe a significantly altered body or organ weights of mice; analysis of total lipids revealed that high proportions of the substrate were incorporated into ether lipids of all organs and tissues during the feeding period, generally accompanied by a remarkable increase in saturated acyl moieties and a concomitant decrease of linoleoyl moieties of total lipids. Yet, four weeks after removal of the ruc-1-*O-*dodecylglycerol-containing diet, the lipids of murine organs and tissues showed a close resemblance to those of the control group. The author, based on the previously reported results, concluded that rac-l-*O-*[1′-14C]dodecylglycerol is converted to common intermediates of ether lipid metabolism in mammals. In this study two major routes for the metabolism were observed: (1) the substrate enters the pathway of ether glycerolipid biosynthesis as a direct precursor and (2) rac-1-*O-*dodecylglycerol as well as ether lipids derived from it, are oxidized by the action of an alkyl monooxygenase. Lauric acid thus formed is not esterified into acyl glycerolipids to an appreciable amount, but further oxidized to acetate and other water-soluble compounds at a fast rate. Only trace amounts of the substrate are excreted in feces. Moreover the author underlined that feeding of a rac-1-*O-*dodecylglycerol-containing diet did not significantly alter body and organ weights in mice.

Haynes *et al*. [69] tested DDG’s bactericidal and antifungal activity against species of *Candida* and *Cryptococcus* which are the major genera causing fungal infections in AIDS patients. All fungi tested were originally isolated from clinical material. From the genus *Cryptococcus*, the species *Cryptococcus neoformans* (six strains, *i.e.*, ATCC 76484 and five isolates from Temple University Hospital [TUH]), *Cryptococcus albidus* (one strain, a TUH isolate), and *Cryptococcus laurentii* (one strain, a TUH isolate) were assayed. From the genus *Candida*, the species *Candida albicans* (one strain [ATCC 32354]), *Candida tropicalis* (two strains, both of which were TUH isolates), and *Candida parapsilosis* (two strains, both of which were TUH isolates) were studied. The authors reported that: (1) the AKG ether rac-1-*O-*dodecylglycerol inhibited the growth of members of two genera of yeasts, *Candida* and *Cryptococcus*, and was strongly synergistic with amphotericin B; (2) at one-half its minimal inhibitory concentration (MIC), DDG decreased the MIC of amphotericin B by as much as 80-fold. This high degree of synergism between DDG and amphotericin B was demonstrated against a number of species of yeasts including *Candida albicans*, *Candida tropicalis*, *Candida parapsilosis*, *Cryptococcus neoformans*, *Cryptococcus albidus*, and *Cryptococcus laurentii*; (3) all fractional inhibitory concentrations, for all strains and species, were calculated to be less than 1, and most were less than 0.6, again demonstrating strong synergism; (4) other AKG ethers with alkyl chain lengths ranging from 8 to 18 carbon atoms were also found to be synergistic with amphotericin B against *C. neoformans* and *C. albicans*; (5) electron microscopy experiments showed that *C. neoformans* grown in the presence of DDG had severely abnormal and deformed capsules; (6) DDG was observed not to simply act as a detergent. The natural detergent sodium deoxycholate, also present in commercial amphotericin B preparations, had no effect at the concentrations of DDG that killed the fungi. So did sodium deoxycholate when it was used in combination with Amphotericin B; (7) DDG did not interact synergistically with the water-soluble antifungal agent fluconazole emphasizing that the lipid-soluble hydrophobic properties of amphotericin B could exert a key role in the synergistic interaction between DDG and amphotericin B; (8) AKG ethers could promote synergism with amphotericin B potentially increasing the interaction between membrane-bound ergosterol and amphotericin B. This study underlines a strong synergistic interaction between DDG and amphotericin B in inhibiting the growth of both *Candida* and *Cryptococcus* species. They hypotized that DDG may act through a mechanism which alters the fluidity of the fungal membrane because it is less hydrophobic than the usual membrane glycerolipids. The Authors observed that the concentrations at which DDG functioned were too low so that it could work as a surfactant, detergent, or emulsifier, which simply dissolved away the lipid-soluble compounds of the membrane resulting in the lysis or rupture of the membrane. This consideration leaded the Author to the hypothesis that the fluidity of the membrane changes sufficiently to alter the hydrophobic environment which changes the activity of key membrane-bound enzymes. When DDG acts synergistically with amphotericin B, it is possible that some of the enzymes, whose activities have been altered because of the membrane fluidity changes, elicited by DDG, are cell wall synthesizing or cell wall-degrading enzymes. amphotericin B kills fungi by binding to the ergosterol in the membrane. The major deterrent to amphotericin B to reach the ergosterol in the membrane is the tight framework of the cell wall. Consequently the authors proposed that a possible mechanism for the synergy observed between DDG and amphotericin B is that DDG is able to act weakening the cell wall because of altered cell wall-synthesizing and/or degrading enzyme activity, then amphotericin B would increase access to the membrane ergosterol. The electron micrograph studies with *C. neoformans* seem to support this hypothesis. This study reports interesting data which are encouraging for the development of new antifungal therapy.

Ved *et al*. [72] treated *S. faecium* ATCC 9790 with 3.5 pg/mL of DDG observing the production of a non-wall entity found in the 25,000× g supernatant cell fraction which activates the autolysin activity of *S. faecium*. The stimulation of the autolysin activity through DDG mimics the activation of the autolysin from a latent to an active form through trypsin and other proteolytic enzymes. The Authors observed that the stimulation of autolytic activity through DDG can be reversed by specific proteinase inhibitors. Moreover DDG also markedly stimulates the proteinase activity endogenous to *S. faecium*, and this stimulation can be reversed by several proteinase inhibitors.

This study shows that DDG does not act directly on the autolysin of the cell wall of *S. faecium*, but rather through an intermediary of cytosolic (or possibly membrane) origin. Since the autolytic activity of *S. faecium* is known to be converted from an inactive to an active form by proteolytic action, it is likely that this intermediary is the proteinase activity endogenous to *S. faecium*. This proteinase can be stimulated by DDG and this stimulation can be reversed by specific proteinase inhibitors which also reverse the stimulation of autolysin activity through DDG. In conclusion, a primary antibacterial mode of action of DDG is to stimulate the proteinase of *S. faecium*, which in turn activates autolysin activity and prevents bacterial growth.

Ved *et al*. [73] designed a study using *S. faecium* ATCC 9790 as primary test organism to assess the effect of DDG on the autolysin activity (0–1,4-*N*-acetylmuramylhydrolase) of this bacterium. This organism has a peptidoglycan hydrolase activity, which catalyzes the cleavage of the p-1,4 linkage between *N*-acetylmuramic acid and *N*-acetylglucosamine of the peptidoglycan. A latent form of this autolysin has been purified to near homogeneity and has demonstrated that it is converted to an active form by proteinases. This study showed that: (1) DDG has a minimum inhibitory concentration of 4 pg/mL compared to 9 pg/mL for 1(3)-dodecanoyl-*sn*-glycerol (monolaurin-dodecanoylglycerol: the fatty acid dodecanoic acid is esterified to glycerol) with *S. faecium* ATCC 9790 as the test organism; (2) the greater potency of DDG can be correlated to its greater retention by the cell; (3) Gram-positive bacteria are more susceptible than Gram-negative bacteria to DDG; (4) the antibacterial action of DDG is not through the physical dissolution of cell walls, but rather as an enzymatic effector; (5) the autolysin activity of whole cells of *S. faecium* is greatly stimulated by DDG; (6) the stimulation of autolytic activity and inhibition of growth respond in parallel to different concentrations of DDG, to DDG *versus* some poorer effector such as monolaurin or a glycerol alkyl ether with a longer or shorter fatty alkyl side chain as dodecanol, and to the antagonistic effects of diphosphatidylglycerol. The Authors, basing on these results, observed that the greater potency of antibacterial activity of DDG can be probably attributed to the fact that ethers are not easily degraded to glycerol and fatty acid as the corresponding esters are. They underlined that the conjugated form of fatty acids, that is fatty acyl glycerol, is known to be a more potent antimicrobial agent than either fatty acid or glycerol alone because the metabolic stability of the ether bond permits the retention of the molecule in a more potent form. Moreover they emphasized that the antibacterial activity of the ethers increased progressively with an increase in the fatty alkyl chain length up to 12 carbons. However, further 2-carbon increases in the chain length caused a decrease in the antibacterial potency of the resulting 1-0-alkylglycerol compounds. This could partly be due to the relative hydrophobic and hydrophilic properties of the different ethers. A hydrophilic nature is necessary for solubility (increased availability) in the suspending medium, and a hydrophobic property facilitates uptake of the compound into the bacterial cell thereby allowing its metabolic effects to be exhibited. The glycerol ether of dodecanol compared to other fatty alcohols probably represents the best balance between hydrophobic and hydrophilic characteristics, and hence it more efficiently brings about the desired antimicrobial effect. In summary: (1) DDG exhibits its antibacterial activity at relatively low concentrations; (2) at these low concentrations, DDG functions metabolically rather than through a direct physical lytic action; (3) DDG stimulates the autolytic activity of *S. faecium*, and the degree of stimulation always occurs under conditions that bring about the same degree of bacterial growth inhibition. Since the proper functioning of the autolysins is critical to the wellbeing of the bacterial cell, it is quite possible that the stimulation of the autolysin could be a primary, but not necessarily the only mechanism by which DDG and related compounds exert their antibacterial activity; (4) DDG does not stimulate the autolysin of *S. faecium* directly (as observed with the segregated cell wall autolysin), but rather through an indirect means which requires the involvement of a membrane- or cytosol-associated entity. The autolysin of *S. faecium* is known to be converted from an inactive to an active form by proteinases.

### 3.7. Other studies involving alkylglycerols

Twenty-five patients with recurrent aphthous stomatitis (RAS) were enrolled in a study designed by Guaranska *et al*. [74] and received treatment with SLO during a three-month period. The frequency of occurrence of RAS decreased from 1.56 before treatment to 0.95 after treatment and the number of lesions per month was significantly reduced during the third month of treatment and two months after treatment; during two months after treatment four patients had no ulcers and an improvement was exhibited in all except three of the remaining patients; a better response of neutrophils to Opsonized Zymosan and PMA were seen; the B cell and T CD3/HLA DR+ cell percentage returned to normal values; a significantly increased percentage of T cells was observed as compared to the previous treatment value; the level of C4 and the hemolytic activity of the complement system decreased after treatment and neared the normal values. This study underlines that SLO has a positive immunomodulation action and is able to alleviate the course of RAS. AKG ability to amplify the PAF biosynthesis in a monocyte cell line, which is known to be produced by mammalian sperm and to be an important activator of sperm motility, was used as a starting point to evaluate the effect of *in vitro* treatment of boar spermatozoa with natural 1-*O-*alkylglycerols (10 mM) on: (1) boar sperm motility; (2) production of PAF and its metabolite, lyso-PAF, by spermatozoa; (3) fertility in artificial inseminations of breeding sows. A computer-assisted spermatozoa analyzer found that

1-*O-*alkylglycerols increased percentage motility as well as velocity parameters after 24 h. These effects were partially or totally reversed by the PAF receptor- antagonist SR 27417. After [^3^H]-1-*O-*alkylglycerol incubation with boar spermatozoa Cheminade *et al*. [75] identified [^3^H]lyso-PAF by high-performance liquid chromatography. Production of PAF and lyso-PAF was measured with a biological assay using [^3^H]serotonin release from rabbit platelets. 1-*O-*alkylglycerols significantly increased lyso-PAF production; 1-*O-*alkylglycerols had no effect on PAF production. The effect of 1-*O-*alkylglycerols on fertilization was also evaluated in industrial breedings: 1-*O-*alkylglycerol treated or untreated semen dilutions were alternately used for artificial inseminations of sows on 12 farms. 1-*O-*alkylglycerol treatment increased the number of farrows but had no effect on the mean size of the litters. In summary, 1-*O-*alkylglycerol treatment of boar spermatozoa *in vitro* improves their motility and fertility; the use of a PAF-receptor inhibitor is able to reverse the effect on motility and the PAF-receptor activation has a key role in the AKG effect on motility.

Zhang *et al*. [76] designed a study to test the hypercholesterolemic activities of pure squalene and SLO in hamsters [five groups (n = 6) of male Golden Syrian hamsters (*Mesocricetus auratus*, 95 ± 5 g)] fed one of five different diets. The diets were: (1) a control diet prepared by mixing the powdered ingredients in the following proportions: casein, 242 g; lard, 50 g; starch, 508 g; sucrose, 119 g; mineral mix, 40 g; vitamin mix, 20 g; and dl-methionine, 1 g; (2) other four diets prepared by adding 0.05, 0.1 and 0.5% pure squalene or 0.05% squalene containing SLO into the control diet. All powered diets were then mixed with gelatin solution (20 g/L) in a ratio of 200 g diet per liter of solution. Once the gelatin had set, the diet was cut into approximately 20 g cubic portions and stored frozen (−20 °C). The authors observed that: (1) there were no significant differences in body weight gain except for the 0.5% squalene supplemented group, which had a final body weight (128 ± 3 g) significantly higher than the control (116 ± 6 g), 0.05% squalene-supplemented group (116 ± 7 g), and 0.1% squalene-supplemented group (112 ± 8 g). For the daily food intake, no significant difference was observed among the control and tested groups; (2) serum total cholesterol concentrations in the experimental groups were observed to be generally higher than the control hamsters. When compared with the control group, total cholesterol was elevated by 32% in 0.05% squalene group, by 23% in the 0.10% squalene group, by 35% in the 0.5% squalene group and by 19% in the 0.05% SLO group. The similar trend was observed for serum triglycerides. However, only 0.05% and 0.5% sualene-supplemented groups had serum total cholesterol concentrations significantly higher than the control (P < 0.05). There was no significant increase in the 0.1% squalene-supplemented and 0.05% SLO-supplemented groups when compared with the control group. High-density lipoprotein cholesterol (HDL-C) was significantly elevated in 0.1% squalene, 0.5% squalene and 0.05% SLO groups but not in the 0.05% SQ group as compared with that in the control hamsters. No significant differences in serum non HDL-C were observed among the five groups; (3) squalene or SLO feeding elevated hepatic cholesterol by 97–133% in the four tested groups compared with the control hamster. In contrast, the cholesterol levels in adipose tissue of the four tested groups remained unchanged as compared with that in the control except 0.5% squalene-supplemented group. No differences in the cholesterol levels of heart were observed among the five groups; (4) the liver squalene concentration in the control group was very low (<0.001 mg/g). Supplementation of squalene in the diet significantly elevated its level in the liver. Supplementation of 0.05% SLO also led to accumulate squalene in the liver to a level that was similar to or higher than the 0.1% squalene group. A similar squalene pattern was also observed in the adipose tissue among the five groups. No differences in heart squalene levels were observed among the control and tested hamsters. This study shows that squalene and SLO are hypercholesterolemic at least in hamsters recommend caution when squalene or SLO are routinely consumed as health supplements.

Burford *et al*. [77] compared various dose levels of batyl alcohol and selachyl alcohol with the known anti-inflammatory treatments aspirin (acetylsalicylic acid) phenylbutazone and hydrocortisone in rats. Both AKG had significant anti-inflammatory activity; the ant-inflammatory effects of the AKG were only effective when administered orally; when given by injection either into the peritoneum or directly into the inflammatory site, they were ineffective; on a dose for dose basis, both AKG were much more effective than aspirin, more potent that phenylbutazone and equally as effective as hydrocortisone when they were both in the lower dose range.

## 4. Conclusions

Sharks seem to be protected from developing cancer, but the reason is yet not understood. SLO is the major source of AKG, glycerol ether lipids which have been widely used in the past years in the Scandinavian medicine because of their properties as immunity boosters and a remedy against radiation therapy and cancer. We have found several clinical trials involving the use of AKG and their ability to bring several benefits to the immunitary system. Among the most interesting properties of AKG, the administration of small amounts (10–100 ng) of these compounds in mice has enhanced macrophage activation for Fc-mediated ingestion activity at the 5th day post treatment [27] and it has partially inhibited PAF-induced platelet stimulation possibly interfering with PAF receptors [30]. Moreover a study shows that a 30 min *in vitro* treatment of peritoneal cells with synthetic DDG has resulted in a greatly enhanced Fc-receptor-mediated ingestion activity of macrophage adherent cells [31]. A study [32] has shown that AKG are able to increase [Ca^2+^]_i_ influx in human Jurkat T-cells possibly by modulating the permeability of calcium channels.

It has been hypothesized that the shark c-myc gene, which is located on the long harm of chromosome 8 in humans, can be inactive or covered [78]. Both reductions of c-myc and inappropriate over expression can be associated with cellular apoptosis and part of the oncogenic process enhancing cell proliferation and inhibition of cell differentiation. The authors did not obtain the sequence for that gene and observed some level of pigment with a background for the sequence. It has also been demonstrated that AKG are able to induce high IL-12 level, a key cytokine for the activation of Th1 responses [34].

In addition the intake of a mixture of CA, BA, M-BA and methoxy-substituted alkyl lipids has been shown to reduce the 5-year cervical cancer mortality and also the radiation-induced neutropenia; the same study has underlined that ether lipids increase blood concentrations of neutrophils and enhance antibody formation [35].

An increased response of neutrophils towards bacteria, an increased level of C4 component of complement in blood, the rise of total antioxidant status of serum, and the predominance of Type 1 cytokine IFN-gamma, TNF-α and IL-2 production by peripheral blood mononuclear cells after SLO intake have also been observed [36]. A study [37] carried out on 10 adult healthy volunteers treated with AKG *per os* for one month, has demonstrated an increase of C1q level and CD4/CD8 ratio from 1.3 to 1.8 and polarized Th1/Th2 lymphocyte cytokine secretion towards Th1. On the contrary no effect on CD4^+^CD25^+^ regulatory lymphocytes was observed; due to the absence of any side effects this innate immunity supporting agent was stated to be safe and effective.

In the oncological area it has been observed that AKG, orally administered to mice affected by Lewis lung carcinoma tumors, have reduced metastasis dissemination by 64 ± 8%, whereas SLO effect has been 30 ± 9% below control [41]. Purified AKG have also decreased significantly plasmalogen content in tumors, whereas SLO has had no such effect. A 5-day treatment with AKG has reduced the presence of von Willebrand factor in tumors emphasizing the anti-angiogenic effect of this compound.

Another experimental study in mice with syngeneic L-1 sarcoma has shown that SLO and fish liver oil together with arctic birch ashes are able to significantly reduce cutaneous angiogenesis induced by tumor cells and tumor growth [42].

The injection of different amounts of SLO in mice at the concentrations of 50 and 10 mg/kg/day, have had maximum DTH response in 48 hours; the percentage of CD8^+^ lymphocytes have increased in the mice injected with SLO; SLO (10 mg/kg/day) injected intraperitoneally has shown a slight non significant decrease of tumor growth rate and in addition SLO injection caused a significant increase in IFN-gamma production [43].

Both human prostate cancer LnCap and DU145 cell lines have responded to the antiproliferative effect of MHG in a similar manner [48].

Furthermore, a clinical trial [66] of radiotherapy for uterine cervical cancers has shown a decrease in cancer growth as well as in the number of both radiation and complex injuries due to AKG pre-radiation treatment; prophylactic AKG administration has dramatically reduced vescical fistulas thus confirming the complementary role of these compounds in supporting the patients against radiotherapy toxicity.

Summarizing, the putative milestones of AKG anticancer effects are mainly their property to activate macrophages and to increase the production of cytokines such as IL-12 and IFN-gamma; IL-12 promotes secretion of IFN-gamma from naive and activated T and NK cells, enhances the cytotoxic activity of NK cells, cytotoxic T lymphocytes, lymphokine activated killer cells and increases the proliferation of preactivated T cells and NK cells [51,52]. The anticancer effect of AKG is also supported by the *O*-alkyl groups storage found into cancer cells that display a low or absent expression of *O-*alkyl monooxygenase enzyme activity as they are unable to face the abnormal distribution of these glyceryl ether lipids [53,54].

Some studies support that AKG could represent, in the future, a new therapeutic agent to overcome the limited access of pharmacological agents for the CNS; in fact it has been shown [58] that intracarotid short-chain AKG constitute a very effective and low toxic strategy for transient opening of the BBB to overcome the limited access of cytotoxic drugs to the brain; nowadays the modern echo- and angiographic techniques represent a simple and safe way to study the carotid artery lumen, and the use of powerful effective toxic chemotherapeutic agents, in a reduced concentration, could be an important therapeutical choice in the management of primary and secondary brain cancers.

This puzzling locoregional approach has never been attempted in humans yet but it is worthy of consideration and attention. Other *in vitro* and experimental investigations using DDG formulations in bacteria and fungi are encouraging for a clinical widespread use against bacterial and fungal infections. Although the studies reported in this review encourage the AKG clinical use, we have not reached the evidence based confirmation yet. Since a study [76] in hamsters has shown that squalene and SLO are hypercholesterolemic, caution should be recommended when squalene or SLO are routinely consumed and more studies should be designed to point out if these effects may occur in human subjects.

Although further randomized clinical trials, involving larger cohorts of patients, are required to finally confirm the properties of these molecules and to definitely exclude possible side effects, a spontaneous anedoctical use of these compounds is strongly recommended, especially on a prophylactic perspective.

## Figures and Tables

**Figure 2:**
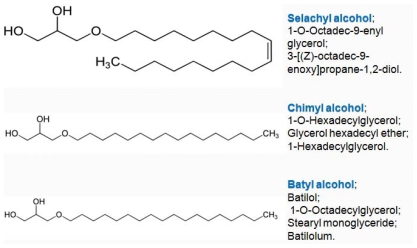
Selachyl, chimyl and batyl alcohols chemical structures.

**Table 1:** AKG are glycerol ether lipids that are naturally occurring in hematopoietic (blood forming) organs such as bone marrow, spleen and liver but they can also be found in neutrophils and in human and cow milk [12,13]. This table shows AKG percentage in human bone marrow, human milk and liver oil. The number of carbon atoms in the first column refers to the long-chain component of the molecule. The number after the colon denotes the number of double bonds. Table adapted from “Biochemical Effects of alkoxyglycerols and their use in cancer therapy” [13].

Alkylglycerols	Human Bone Marrow	Human Milk	Greenland Shark Liver Oil
14:0			2.0
15^a^			0.7
16:0	29.4	23.9	9.1
16:1		Trace	10.8
17^a^	7.6	3.6	3.6
18:0	24.6	22.8	2.8
18:1	16.7	33.8	59.4
18:2		1.4	1.6
18:3			?
19^a^	6.1	2.4	1.5
20:0	2.9	1.6	
20:1	3.2	2.3	6.2
22:0	0.7	0.7	
22:1	5.1	3.4	2.2
24		2.1	

a Both branched and normal chains C_15_, C_17_, and C_19_ are present.

## Abbreviations

AKG: alkylglycerols
APC: alkylphosphocholine
AA: arachidonic acid
bFGF: basic fibroblast growth factor
BBB: blood-brain barrier
CEA: Carcino-EmbryonalesAntigen
C-injuries: complex injuries
CK: creatine kinase
CNS: central nervous system
DAG: diacylglycerol
DDG: dodecylglycerol
DTH: delayed-type hypersensitivity
EA: serum frec-01% egg albumin supplemented RPMI medium
ErPC: erucylphosphocholine
ET-16-OCH3: 1-O-hexadecyl-2-metoxyglycero- 3-phosphatidylcholine
FCS: fetal calf serum
FITC: fluorescein isothiocyanate
fMLP: formyl peptide
i.v.: intravenous injection
HDL-C: high-density lipoprotein cholesterol
HG: sn-1-O-hexadecylglycerol
I-injuries: total number of injuries
MDA: malondialdehyde
MHG: 1-O (2 methoxy) hexadecyl glycerol
MIC: minimal inhibitory concentration
MTX: methotrexate
NK: natural killer
NO: nitric oxide
PAEC: pulmonary arterial endothelial cells
PAF: platelet activating factor
PMA: phorbol-12-myristate-13-acetate
PPS-C16 ODN: phosphorothioate-protected oligonucleotides with 1-O-hexadecylglycerol
PUFA: polyunsatured fatty acids
SLO: shark liver oil
[Ca2+]i: calcium concentrations
RAS: aphthous stomatitis
R-injuries + C-injuries: sum of the injuries
ROS: reactive oxygen species
